# Supplementary material for: High-Risk Obesity Phenotypes: Target for Multimorbidity Prevention at the ROFEMI Study
Source: J Clin Med. 2022 Aug 9;11(16):4644. doi: 10.3390/jcm11164644 (PMC9410284; doi:10.3390/jcm11164644)
Supplement: Supplementary file 1 [file jcm-11-04644-s001.zip › jcm-1803132-supplementary.pdf]

Table S1. Qualitative Variables by Groups

Variable (Absolute number (percentage))	Group 1 n=74	Group 2 n=462	*p*
Sex (women)	58 (78.4)	216 (46.7)	***0.000***
Smoker	9 (12.2)	72 (15.6)	0.44
Education level	(n=72)	(n=453)	
Illiterate	3 (4.2)	33 (7.3)	
Primary	41 (56.9)	190 (41.9)	
Secondary	19 (26.4)	170 (37.5)	0.10
University	9 (12.5)	60 (13.2)	
Employed	27 (36.5)	177 (38.4)	0.7
Origin (Urban)	50 (67.6)	349 (75.5)	0.14
Physical activity	19 (25.7)	157 (33.9)	0.15
HBP	49 (66.2)	331 (71.6)	0.33
T2DM	22 (29.7)	199 (43.1)	***0.03***
Dyslipidemia	41 (55.4)	313 (43.1)	***0.03***
Hyperuricemia	2 (2.7)	102 (22.2)	***0.0001***
HFpEF	9 (12.2)	69 (14.9)	0.39
HFrEF	1 (1.3)	19 (4.1)	0.39
CAD	8 (10.8)	42 (9.1)	0.63
Stroke	5 (6.8)	35 (7.6)	0.8
Gastroesophageal reflux disease	16 (21.6)	67 (14.5)	0.11
COPD/Asthma	7 (9.5)	48 (10.4)	0.8
Cancer	1 (1.35)	13 (2.8)	0.46
Arthrosis	29 (39.2)	141 (30.5)	0.14
Depression	17 (22.9)	98 (21.3)	0.73
Disability			
Moderate	13 (17.6)	93 (20.3)	0.8
Severe	6 (8.1)	42 (9.15)	0.8
Previous treatment			
Glucocorticoids	5 (6.8)	28 (6.1)	0.82
Metformin	17 (22.9)	156 (33.9)	0.06
Sulfonylureas	4 (5.4)	9 (1.9)	0.07
DPP-4 inhibitors	4 (5.4)	38 (8.3)	0.39
GLP-1 RA	6 (8.1)	78 (16.9)	0.05
SGLT2 inhibitors	9 (12.2)	79 (17.2)	0.27
Insulin	12 (16.2)	54 (11.7)	0.27
Statins	35 (47.3)	259 (56.3)	0.14
IBP	45 (60.8)	231 (50.2)	0.09
Antihypertensives	47 (63.5)	325 (70.8)	0.2
NSAIDs	17 (22.9)	65 (14.2)	0.05
Antidepressants	21 (28.4)	104 (22.6)	0.28

Legend: CAD: coronary artery disease; COPD: chronic obstructive pulmonary disease; DPP-4 inhibitors: dipeptidyl-dipeptidase 4 inhibitors; GLP-1 RA: glucagon like peptide-1 receptor agonist; HBP: high blood pressure; HFpEF: heart failure with preserved ejection fraction; HFrEF: heart failure with reduced ejection fraction; IBP: protons bomb inhibitors; NSAIDs: nonsteroidal anti-inflammatory drugs; SGLT2 inhibitors:

sodium-glucose cotransporter 2 inhibitors; T2DM: type 2 diabetes mellitus. Data are expressed by absolute number and percentage.

Table S2. Quantitative Variables by Groups

Variable (median/interquartile range)	Group 1 n= 74	Group 2 n=462	*p*
Age (years)	61 (27)	62 (22)	0.87
Weight (Kg)	79.9 (11)	97 (22)	***0.0000***
BMI (Kg/m^2)	31.3 (3.7)	34.7 (6.9)	***0.0000***
WC (cm)	97.5 (10)	112 (13.5)	***0.0000***
Charlson	1 (2)	1 (3)	***0.002***
FPG (mg/dL)	100 (26)	104 (32)	***0.04***
HbA1c (%)	5.8 (0.7)	5.9 (1.3)	0.32
eGFR (ml/min/1.73m^2)	86.3 (27.3)	84.1 (36)	0.29
Uric acid (mg/dL)	5 (1.8)	5.8 (2.5)	***0.0000***
hsCRP (mg/dL)	2 (4)	3 (5.5)	0.36
LDL-c (mg/dL)	107 (60)	97 (52)	0.57
HDL-c (mg/dL)	52 (17)	46 (16)	***0.0001***
Triglycerides (mg/dL)	114 (72)	136 (86)	***0.002***
TyG index	8.6 (0.8)	9.6 (0.7)	***0.003***
AST (U/L)	22 (17)	22 (17)	0.6
ALT (U/L)	21.5 (20.5)	21 (12.7)	0.56
GGT (U/L)	25 (47.5)	32 (31)	0.07
ALP (U/L)	81.5 (42)	79 (35)	0.53
Hemoglobin (g/dL)	13.7 (2)	14 (2.3)	***0.001***
Leukocytes (x10^9/L)	6.8 (2.97)	7.4 (2.8)	***0.01***
Lymphocytes (x10^9/L)	2.12 (1.2)	2.13 (1.1)	0.53
Platelets (x10^9/L)	228 (82)	237 (97)	0.33
Albumin (g/dL)	4.2 (0.5)	4.3 (0.5)	0.06
UACR (mg/g)	11.1 (17.1)	9.4 (19.4)	0.8
Drugs number	6 (6)	7 (6.25)	***0.79***

Legend: ALP: alkaline phosphatase; ALT: Alanine transaminase; AST: Aspartate transaminase; BMI: body mass index; eGFR: estimated glomerular filtration rate; FPG: Fasting plasma glucose; GGT: gamma-glutamyl transferase; HbA1c: glycated hemoglobin; HDL-C: high density lipoprotein cholesterol; hsCRP: high-sensitivity C-reactive protein; LDL-C: low density lipoprotein cholesterol; TyG index: triglyceride-glucose index; UACR: Urinary albumin-to-creatinine ratio; WC: waist circumference. Data is expressed as median and interquartile range since non normality of the data.
